# Supplementary material for: A retrospective cohort study evaluating the association between opioid and alcohol-related emergency department presentations and the subsequent risk of hospitalization
Source: PLoS One. 2025 Jun 11;20(6):e0325083. doi: 10.1371/journal.pone.0325083 (PMC12157778; doi:10.1371/journal.pone.0325083)
Supplement: Attachment — Submitted filename: Response_to_reviewers_comments_17.04.25.docx [file pone.0325083.s002.docx]

**Response to reviewers – Round 1**

A retrospective cohort study evaluating the association between opioid and alcohol-related emergency department presentations and the subsequent risk of hospitalization

**Editors Comments:**

**This study highlights a very important problem in the current medical scenario. There is a big disparity in the long-term management of patients with OUD and a high risk of increased healthcare utilization. This is a well-conducted study. Are there similar studies that looked into these two conditions and hospital admissions? If so, can you provide a background in the introduction section? Please refer to the questions brought on by the reviewers.**

- Thank you for your comment, previous studies have been highlighted in the introduction starting line 94.

**Can you comment on strategies to engage patients for long-term outpatient care - starting opioid management at the time of hospital discharge and linkage with outpatient primary care and opioid management? One of the studies that looked into this is the NavSTAR trial which showed that patient navigation could reduce hospital admissions amongst patients with comorbid SUDs.**

**(**[**https://doi.org/10.1186/s13722-024-00463-9**](https://doi.org/10.1186/s13722-024-00463-9)**)**

- Thank you for your suggestion, please see the added section starting line 327.

**The study has identified some key findings including social determinants that may affect the readmissions in ED including homelessness. Strategies to address this issue can be included in the conclusion section.**

- Thank you for your comment, please see revision starting line 375.

**Reviewer #1**

**Thank you for this important and timely study.**

**I would suggest that utilizing more recent references would strengthen your manuscript as many in the introduction are very old, especially 1, 2, 5, 6, 24. More current relevant references on opioids can be found in the 2024 CRISM guideline for managing opioid use disorder (CMAJ 2024 November 12;196:E1280-90. doi: 10.1503/cmaj.241173) and CCSA reports.**

- Thank you for your suggestion, more up to date references have been added in the introduction and throughout the paper to strengthen the overall manuscript.

**The first description triage as a variable is in the results section and I suggest providing some introduction to this variable earlier on.**

- Thank you, a section introducing triage as a variable has been added on line 163.

**Please also pay more attention to formatting of the references and manuscript text.**

- Thank you, referencing has been standardized throughout the manuscript using Vancouver style referencing.

**I wonder if including the 320 patients with both opioid and alcohol presentations in the opioid group has skewed the results and suggest excluding those patients or at least running subgroup analyses without them.**

- Thank you. We chose to default patients to the opioid group and adjust for alcohol use because it opioid use typically poses higher immediate risks, such as overdose, and is the primary focus of treatment with medications like opioid agonist therapy. This classification reflects the clinical emphasis on managing opioid use disorder, especially when opioid-related complications are more pronounced than alcohol use.

**Reviewer #2**

**Recommendations:**
**- Provide a clear and concise statement of your research questions and hypotheses in the introduction.**

- Thank you for your recommendation, please see an addition to the introduction clearly highlighting our research statement and hypotheses.

**- Elaborate on your data collection and data de-identification process in the method section.**

- Thank you, this was added on line 123.

**- Ensure your research questions and hypotheses are consistently addressed in the data analysis plan and results sections.**

- Thank you for your recommendation, added sections starting line 186 and line 191 have been added.

**-Finally, clarify the rationale for covariate selection and specify which were included or controlled for in the regression models.**

- Thank you this was added on line 175.

  **Overall Evaluation:**
  **This manuscript offers important contributions to understanding the healthcare impact of OUD compared to alcohol use disorder. By strengthening the articulation of the study's research questions, hypotheses, data analysis plan, and the rationale behind methodological choices, the authors can improve the clarity, focus, and overall impact of the work.**
- We sincerely appreciate your detailed and insightful review.

**Reviewer #3: Dear author, despite of the study design and limitations explained in the article. This thematic is absolutely necessary to understand this population and their needs. I have no suggestion to methodology however you have to identify the bias and how they have impacted in the results.**

- Thank you for your comment, a section has been added on line 348 to identify the potential bias, and their impact on results.
